# Bioremediation of Wastewater by Iron Oxide-Biochar Nanocomposites Loaded with Photosynthetic Bacteria

**DOI:** 10.3389/fmicb.2017.00823

**Published:** 2017-05-23

**Authors:** Shiying He, Linghao Zhong, Jingjing Duan, Yanfang Feng, Bei Yang, Linzhang Yang

**Affiliations:** ^1^Institute of Agricultural Resources and Environment, Jiangsu Academy of Agricultural SciencesNanjing, China; ^2^Department of Chemistry, Pennsylvania State University, Mont Alto, PAUnited States

**Keywords:** iron oxide nanoparticles, nanocomposites, microorganisms, biochar, nutrient removal, water treatment

## Abstract

It has been reported that bacteria-mediated degradation of contaminants is a practical and innocuous wastewater treatment. In addition, iron oxide nanoparticles (NP) are wastewater remediation agents with great potentials due to their strong adsorption capacity, chemical inertness and superparamagnetism. Therefore, a combination of NPs and microbes could produce a very desirable alternative to conventional wastewater treatment. For this purpose, we first prepared Fe_3_O_4_/biochar nano-composites, followed by loading photosynthetic bacteria (PSB) onto them. It was found that Fe_3_O_4_/biochar nano-composites exhibited a high adsorption capacity for PSB (5.45 × 10^9^ cells/g). The efficiency of wastewater pollutants removal by this PSB/Fe_3_O_4_/biochar agent was then analyzed. Our results indicated that when loaded onto Fe_3_O_4_/biochar nano-composites, PSB’s nutrient removal capability was significantly enhanced (*P* < 0.05). This agent removed 83.1% of chemical oxygen demand, 87.5% of NH_4_^+^, and 92.1% of PO_4_^3-^ from the wastewater in our study. Our experiments also demonstrated that such composites are outstanding recyclable agents. Their nutrient removal capability remained effective even after five cycles. In conclusion, we found the PSB/Fe_3_O_4_/biochar composites as a very promising material for bioremediation in the wastewater treatment.

## Introduction

Water pollution remains as an enduring environmental problem that accompanies with worldwide human population increase and economic development. The organic pollutants, excessive phosphorous (P) and nitrogen (N) released through runoff, frequently result in the eutrophication of water bodies, which is harmful to the health of human beings and to the ecological environment (Beaver et al., 2014; Kirkpatrick et al., 2014; Li et al., 2014). It is desirable to establish environmentally benign and economically cost-effective measures to keep such pollutions under control. Significant efforts have already been made to improve wastewater treatment with different approaches, such as adsorption (Vecino et al., 2014; Douglas et al., 2016), coagulation (Zhu et al., 2016), photocatalytic oxidation (Chong et al., 2010), and biodegradation (Wu et al., 2010, 2011; Li et al., 2016). Unfortunately, their effectiveness in actual applications are frequently hampered by various factors (Oller et al., 2011) such as energy, efficiency, stability, and economy. Each approach has its own pros and cons, and none has the merits in all aspects. Nevertheless, scientists have never stopped looking for practices that can improve water treatment.

The combination of biodegradation and nanotechnology is suggested as a potential efficient, low-cost and environmental benign technique (Huang et al., 2014; Oh et al., 2016). Human has a long history of biological wastewater treatment by microorganisms. This worldwide approach has been proven as an effective and environmental-friendly strategy. The metabolic diversity of microorganisms ensures a variety of substrates to be consumed. Therefore, applications of bacteria such as *Pseudomonas aeruginosa* (Shukla et al., 2014), *Aspergillus niger* (Vassilev et al., 1997), and *Rhodopseudomonas sphaeroides* (Liu et al., 2015) in wastewater treatment have been investigated. It is well known by now that they can degrade toxic pollutants in aqueous media during their metabolisms. Amongst, photosynthetic bacteria (PSB) have been found as an effective and eco-friendly species that can simultaneously remove carbon, nitrogen and phosphorous in the synthetic sewage and soybean wastewater treatments (Nagadomi et al., 2000; Lu et al., 2010; Idi et al., 2015). On the other hand, limitations of biological wastewater treatment are apparent at the same time. Such biodegradation processes are usually slow. It is difficult to recover cells, and activities of recovered cells are significantly inhibited by substrates. Consequently, its application is greatly restricted.

The goal of our investigation is develop a system that couples biodegradation and nanotechnology with enhanced activity and stability of the biocatalyst, and with a desirable recycling property (Lenz et al., 2009; Li et al., 2015). Iron oxide nanoparticle (NP) has novel properties such as strong adsorption capacity, chemical inertness, high biocompatibility and superparamagnetism. Besides, NP amendment to enhance microbial metabolic activity has gained increased attentions in the recent years due to unique surface and quantum size effects of NP (Pan et al., 2010; Stone et al., 2010). These appealing features allow their applications as microbial immobilization carrier to enhance biocatalytic efficiency. For example, Xu et al. (2011) has successfully used iron oxide NP as a cell immobilization carrier with minimal mass transfer resistance. Gadhe et al. (2015) took advantage of NP’s high electron transfer rate to boost microbial enzyme activity. Moreover, NP alone has been applied in contaminant controls. For example, inexpensive iron oxide NP has been employed as effective nanosorbents for the removal of a broad range of environmental contaminants such as metal ions (Liu et al., 2008) or dye (Iram et al., 2010). They have also been exploited as catalysts for the degradation of 4-chlorophenol (Xu and Wang, 2012). However, all these explorative endeavors face a common and major setback that, spontaneous aggregations in iron oxide NP solution quickly annihilate their function. To prevent aggregations from happening, one approach is to load NP onto carriers. Biochar is created from various biomass materials including agricultural and forestry residues through anaerobic pyrolysis. The large effective surface area, high porosity and abundant functional groups (Zhang et al., 2012) make it a perfect carrier for NP. Its wide application can also benefit from low cost, being eco-friendly, and most importantly, its ubiquitous availability. For example, Yao et al. (2013) has used biochar as an effective adsorbent in waste removal. Naturally, biochar is chosen as the most ideal carrier for iron oxide NP in our study.

Besides to find the features of PSB/NP/biochar composite, another goal for our study is to develop a novel and effective technique in waste water treatment. We hope this composite can utilize advantages from individual components of, and a combination of, iron oxide NP (for adsorption, catalysis and magnetic separation), PSB (for biodegradation) and biochar (for adsorption). For these purposes, we first prepared multifunctional Fe_3_O_4_/Biochar nanocomposites, followed by immobilizing PSB, *R. capsulatus*, onto their surfaces. The physical and chemical characterizations of Fe_3_O_4_/Biochar, with and without *R. capsulatus*, were conducted. We measured products’ capability in simultaneously removing chemical oxygen demand (COD), ammonia (NH_4_^+^), and phosphate (PO_4_^3-^) in aqueous solutions. Their regeneration property, one of the important characteristics for waste water treatment, was studied up to five recycles.

## Materials and Methods

### Bacteria Strain and Cultivations

The strain of PSB bacteria, *R. capsulatus*, a purple non-sulfur bacterium, used in experiments was isolated in our laboratory and cultured in the medium containing purvate, yeast extract, NaCl, NH_4_Cl, MgCl_2_, and K_2_HPO_4_ at pH 7 and 30°C under continuous illumination with incandescent lamps at a light intensity of about 2000 lux. All solutions were made up with sterile deionized water.

### Synthesis of Fe_3_O_4_/Biochar

Wheat straw was used to produce biochar through slow pyrolysis using a pyrolyzer at temperatures of 500°C continuously flushed with N_2_. And the temperature was maintained for 2 h. Then the biochar was allowed to cool to room temperature, and sieved through a 0.15-mm mesh. Then biochar was pretreated with 0.1 M HNO_3_ for 4 h, washed with distilled water and then dried at room temperature. Fe_3_O_4_ NPs were synthesized by conventional chemical coprecipitation method (Molday, 1984). Briefly, a solution of FeCl_3_ and FeSO_4_ mixture (molar ratio 2:1) was prepared, followed by dropping enough aqueous NH_4_OH solution with vigorous stirring for 30 min under N_2_ protecting. The generated Fe_3_O_4_ NPs were filtered and washed three times with distilled water by magnetic separation. The Fe_3_O_4_/biochar was prepared as follows: the obtained Fe_3_O_4_ (about 0.5 g) were diluted to 500 mL deionized water. Subsequently, 2 g biochar were introduced to the Fe_3_O_4_ suspension and the mixture was stirred for 4 h at room temperature. The Fe_3_O_4_/biochar composite obtained was washed immediately with distilled water for five times by magnetic separation and dried in an oven at 80°C for 6 h.

The products were characterized by field emission gun scanning electron microscopy (SEM, JEOL, JSM-5610LV) and transmission electron microscopy (TEM, JEOL, JEM-200CX). For TEM analysis, 5 μL sample was placed onto a carbon-coated copper grid and allowing the solvent to evaporate in air.

The crystal structures were determined using X-ray diffraction (XRD) (Shimadzu XD -3A). The patterns with the Cu Karadiation (*k* = 1.54051 Å) at a generator voltage of 40 kV and current of 40 mA were recorded in the region of 2𝜃 from 10° to 70°. Brunauer–Emmett–Teller (BET) surface area of the samples was determined by using a computer-controlled nitrogen gas adsorption analyzer (Quantachrome-NOVA4000e). FTIR spectra of the materials were recorded with KBr disks in the range of 4000–400 cm^-1^ on Nicolet Magna FTIR-750 spectrometer. The zeta potential (ζ) of the Fe_3_O_4_ NP and Fe_3_O_4_/biochar was measured with a zeta potential analyzer (BECKMAN, Delsa 440SX).

### Immobilization of Bacteria with Fe_3_O_4_/Biochar Composite

The PSB, *R. capsulatus*, was cultivated in medium for 72 h at 30°C. Cells were harvested at the mid-log phase of the growth curve, as shown by an optical density of 0.3–0.5 at 600 nm (OD_600_), by centrifugation (5000 rpm) at 4°C for 10 min. The collected bacteria were washed five times with distilled water and then re-suspended in distilled water. Then 1 g magnetic Fe_3_O_4_/biochar was mixed with 1 g cell (wet weight) in 500 mL culture solution. In order to make the Fe_3_O_4_/biochar and cells contact sufficiently, the flask was placed in the shaker for 2 h at 30°C until cells adsorbed on the surface and pores of Fe_3_O_4_/biochar. A permanent magnet was applied to the flask, separating the immobilized biomass from mixture. The immobilized cells were washed gently two times with distilled water to remove free cells. Bacteria supported on Fe_3_O_4_/biochar so obtained were used to study the nutrient (COD, NH_4_^+^, and PO_4_^3-^) removal in water.

The surface morphologies of Fe_3_O_4_/biochar supported *R. capsulatus* were determined by SEM. The amount of PSB cells on Fe_3_O_4_/biochar were measured by qPCR assays. Genomic DNA was subsequently extracted using bacterial DNA kit (Omega), following the manufacturer’s instruction. The copy numbers of *puf*M gene were quantified by real-time qPCR analysis (C1000^TM^ Thermal Cycler equipped with CFX96^TM^ Real-Time system) using primer *puf*M.557F/750R (Feng et al., 2014). The qPCR standard curve was generated as described by Feng et al. (2011). The 25 μL reaction mixture contained 12.5 μL of SYBR Premix Ex Taq ^TM^, 0.5 μM of each primer, 0.5 μL of BSA at 20 mg/mL initial concentration, and 1.0 μL template containing approximately 2–9 ng DNA. Same procedure was carried out for a blank of using water as the template. The amplicons were confirmed by agarose gel electrophoresis of qPCR amplicons of the *puf*M gene and the melting curve analysis always resulted in a single peak. Real-time PCR was performed in triplicate, and the high amplification efficiencies of 97.4–104% were obtained with high consistency *R*^2^ values of 0.976–0.997.

### Nutrient Removal Efficiency

For the treatment experiment, the chemical composition of the synthetic wastewater was as follows: glucose, NH_4_Cl, KH_2_PO_4_, MgSO_4_⋅7H_2_O, NaHCO_3_, CaCl_2_⋅2H_2_O. The initial COD, NH_4_-N, and TP of the wastewater were 877 mg/L, 29.7 mg/L, and 3.6 mg/L, respectively. Batch experiments were carried out in a 250 mL Erlenmeyer flask as bio-reactor at room temperature with a light intensity of 2000 lux.

The Fe_3_O_4_/biochar composite (0.1 g), free cells (the same amount of immobilized) and PSB/ Fe_3_O_4_/biochar [immobilized PSB (approx. 0.05 g wet weight) and Fe_3_O_4_/biochar (0.05 g)] were mixed with 100 mL synthetic wastewater, respectively. The wastewater without any agents was set as control. Then, the suspension was sampled from the bioreactors by magnetic separation at different periods. The supernatant was filtered through a 0.45 μm membrane filter to test the concentrations of nutrient including COD, NH_4_^+^, and PO_4_^3-^. COD was measured using a COD analyzer (DR1010 COD, HACH, China). The phosphate concentrations in the samples were analyzed by molybdenum blue spectrophotometric method (Shimadzu spectrophotometer UV-3150PC). The concentrations of NH_4_^+^ were determined by the Nessler reagent colorimetric method. The numbers of PSB during nutrient removal were measured by qPCR.

Upon completion of the wastewater reduction process, the cells were recovered by magnetic separation, and then washed three times by deionized water. The fresh wastewater was added to the flask in consecutive degradation processes to test the reusability of PSB/Fe_3_O_4_/biochar agent under the same conditions. Each experiment was conducted in triplicate.

### Statistical Analysis

Statistical procedures were performed with the SPSS 13.0 software package for Windows. All the experiments were done in triplicate, and the data were expressed as the means with standard deviation (SD). The significance of the difference between the treatments means was assessed by Tukey’s multiple range tests. Differences at *P* < 0.05 were considered statistically significant.

## Results and Discussion

### Characteristics of Fe_3_O_4_/Biochar Nanocomposite and Immobilized *R. capsulatus*

**FIGURE 1:**
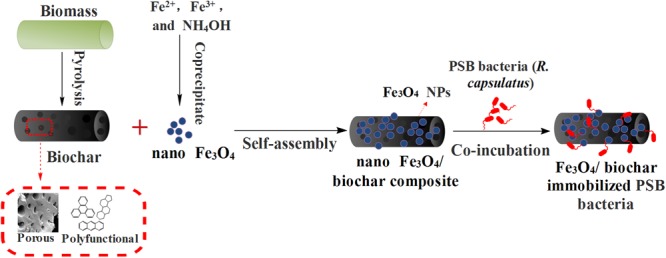
**Scheme of preparation of Fe_3_O_4_/biochar composite and the immobilization of photosynthetic bacteria (PSB) bacteria**.

**FIGURE 2:**
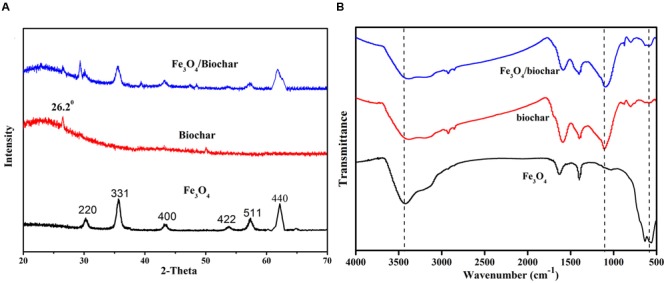
**(A)** X-ray diffraction (XRD) patterns and **(B)** FTIR spectra of Fe_3_O_4_, biochar, and Fe_3_O_4_/biochar.

**Figure 1** illustrates our product preparation process. Biochar prepared from biomass pyrolysis has a large surface area. It provides numerous functional groups for Fe_3_O_4_ NPs to tether at. A good mixing ensures the formation of Fe_3_O_4_/biochar nanocomposite. To characterize their morphologies, we collected and compared X-ray diffraction patterns of Fe_3_O_4_, biochar and Fe_3_O_4_/biochar (**Figure 2A**). Main diffraction peaks for the Fe_3_O_4_, corresponding to the (220), (311), (400), (422), (511), and (440) planes, match well with crystalline cubic Fe_3_O_4_ (JCPDS: 65-3107). All these peaks appear again in the spectrum for Fe_3_O_4_/biochar nanocomposite, suggesting the crystal structure of Fe_3_O_4_ is retained. Crystalline Fe_3_O_4_ are in both samples, indicated by the strong and sharp peaks. The peak at 26.2° is present in both biochar and nanocomposite. Its relatively high intensity and symmetry are a result of graphitization. Furthermore, the broad band at 20–30° indicates that majority of biochar remains as amorphous in the nanocomposite.

Studies of Fe_3_O_4_ NP adsorption onto different materials have been reported. Due to the intrinsic nature of biochar, Fe_3_O_4_ particles can adsorbed to the biochar FTIR has been a useful tool to confirm the adsorption. We collected FTIR spectra for Fe_3_O_4_ NP, biochar, and Fe_3_O_4_/biochar nanocomposite (**Figure 2B**). The spectrum for nanocompsite largely resembles that for biochar. However, the key peak at 636 cm^-1^, ascribed to Fe–O stretching, is observed in the nanocomposite spectrum. This observation reflects the presence of iron oxide in the composite.

**FIGURE 3:**
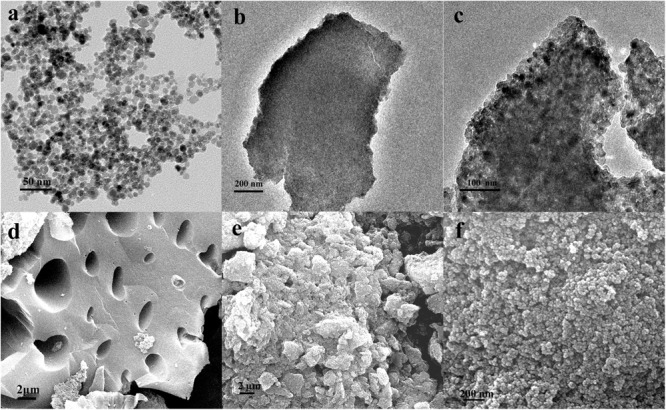
**TEM micrographs of (a)** Fe_3_O_4_ nanoparticles (NPs), **(b)** biochar, and **(c)** Fe_3_O_4_/biochar, SEM images of **(d)** biochar, **(e)** Fe_3_O_4_/biochar, and **(f)** magnified SEM image of Fe_3_O_4_/biochar.

We examined the morphology and size of the obtained materials with TEM and SEM techniques. TEM image of Fe_3_O_4_ shows that these NPs are spherical, with an average diameter of 10 ± 2.5 nm (**Figure 3a**). With SEM images, we found the surfaces of biochar are rough and porous (**Figure 3d**), providing large contact surfaces to for Fe_3_O_4_ particles to deposit onto. Fe_3_O_4_ particles are uniformly dispersed with very little aggregation in TEM (**Figures 3b,c**) and SEM (**Figures 3e,f**) images.

The data of BET surface area, pore size diameters and pore volume of the materials were measured and presented in **Table 1**. The specific surface area of the as-prepared Fe_3_O_4_/biochar was 114.85 m^2^/g. It is interesting to notice this area is close to the sum of areas for Fe_3_O_4_ (67.12 m^2^/g) and for biochar (42.08 m^2^/g). Nevertheless, the greatly increased surface area proves the Fe_3_O_4_ are tethered onto biochar surfaces.

**Table 1:** Brunauer–Emmett–Teller (BET) analysis of biochar, Fe_3_O_4_, and Fe_3_O_4_/biochar.

Samples	Biochar	Fe_3_O_4_	Fe_3_O_4_/biochar
Specific surface area (m^2^/g)	42.08	67.12	114.85
Pore size diameters (nm)	4.34	–	5.78
Pore volume (cm^3^/g)	0.27	–	0.39

**FIGURE 4:**
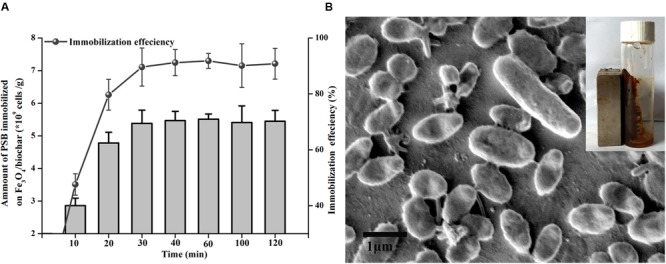
**(A)** The amount of immobilized PSB bacteria (bar), as well as the immobilization efficiency (line), on Fe_3_O_4_/biochar, and **(B)** SEM image of immobilized PSB bacteria, the insert shows the magnetic property of immobilized bacteria.

An increased surface area of Fe_3_O_4_/biochar nanocomposite accommodates abundant active sites for bacteria to attach onto as shown in **Figure 1**. We measured the amount of immobilized bacteria with qPCR. As shown in **Figure 4A**, the Fe_3_O_4_/biochar exhibits an excellent adsorption performance on PSB. The adsorption is fast, and the efficiency plateaus at about 90% after 30 min of incubation. Its adsorption capacity is found to be approximately 5.45 × 10^9^ cells/g. SEM images show that cells are adsorbed onto the surface of Fe_3_O_4_/biochar composite, and the morphologies of immobilized bacteria appear to remain unchanged (**Figure 4B**). We believe the exceptional adsorption phenomenon roots from several reasons. First, Fe_3_O_4_/biochar nanocomposite offers a larger specific surface area, higher surface energy, and hence more accessible active sites. When these factors are combined, it is not a surprise that this nanocomposite has an increased efficiency in the microbial cells adsorption. Secondly, electrostatic interactions play a facilitating role in adsorption. The outer membrane of PSB cell wall consists of negatively charged lipopolysaccharides and phosphate. At the same time, the zeta potential values of Fe_3_O_4_ and Fe_3_O_4_/biochar were 14.8 and 4.2 mV, respectively, suggesting their surfaces is mostly positively charged. Strong electrostatic attractions ensure an enhancement in the adsorption. Moreover, the adsorption is facilitated by the hydrophobic interactions between constituents of bacteria (e.g., proteins, lipopolysaccharides, mycolic acid, and etc) and surface of biochar.

Having magnetic Fe_3_O_4_ in the final product leads to an important feature that distinguishes it from other similar products. When placed next to a magnetic bar, the Fe_3_O_4_/biochar with immobilized PSB can be easily separated by the external magnetic field (insert of **Figure 4B**). This feature allows an extremely convenient, and more importantly, non-destructive, separation method for this product. A wide range of applications can take advantage of this feature by using Fe_3_O_4_/biochar nanocomposite as the support for PSB.

### Nutrient Removal

We developed the Fe_3_O_4_/biochar nanocomposite, as well as the subsequent PSB/Fe_3_O_4_/biochar composite, for water treatment. Therefore, their efficiencies in being sorbent and/or catalyst were evaluated in our study. The results are presented below.

#### Removal of COD

**FIGURE 5:**
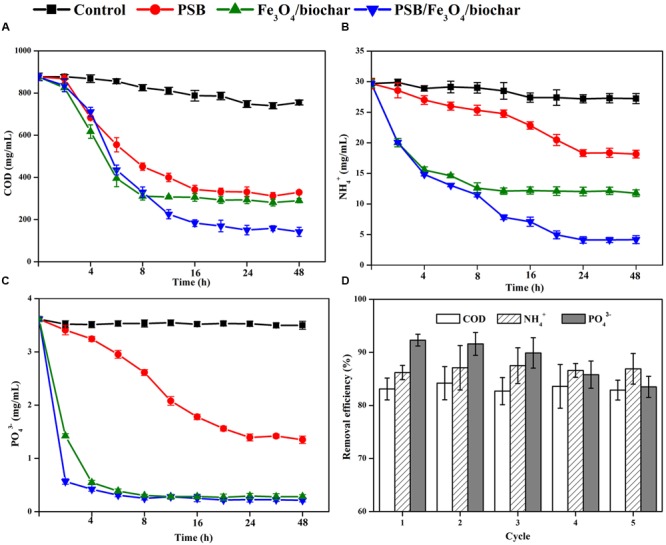
**Removal of (A)** COD, **(B)** NH_4_^+^, and **(C)** PO_4_^3-^ (by PSB, Fe_3_O_4_/biochar, and PSB/Fe_3_O_4_/biochar). **(D)** Nutrient removal ability of PSB/Fe_3_O_4_/biochar during five cycles.

**FIGURE 6:**
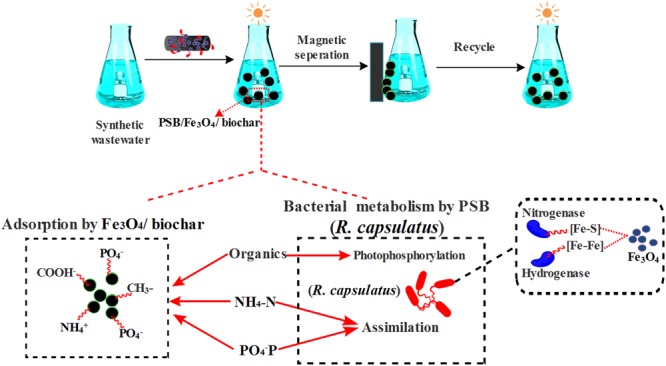
**Possible nutrient removal mechanisms by the PSB/Fe_3_O_4_/biochar**.

It is well known that PSB can catabolize organic species through photophosphorylation by utilizing light as the additional energy source. After 30 h of treatment, 62% of COD in synthetic wastewater were removed by free PSB bacteria (**Figure 5**) The narrow 2.2-eV band gap of Fe_3_O_4_ NPs (Akhavan and Azimirad, 2009) makes them effective photocatalysts for absorbing visible light. The Fe_3_O_4_/biochar nanocomposite has a high mesopore volume, a larger surface area and multiple sites for PSB adsorption. Because of such properties that favor adsorption and light degradation, Fe_3_O_4_/biochar nanocomposite (0.1 g) exhibits 67% COD removal. When PSB (approximately 0.05 g) are immobilized onto such nanocomposite (0.05 g), COD removal efficiency is boosted to 83%. This represents a ca. 50% enhancement, compared to PSB only, or Fe_3_O_4_/biochar nanocomposite only. This Fe_3_O_4_/biochar nanocomposite performs a better efficiency on COD removal than that of constructed wetlands methods (∼65% COD) (Wang et al., 2016) and has a similar removal efficiency as the active sludge system does (60–90% COD) (Nagadomi et al., 2000; Eldyasti et al., 2011). A proposed mechanism for this enhancement is illustrated in **Figure 6**. First, the PSB *R. capsulatus* is tightly trapped on the Fe_3_O_4_/biochar composite with high capacity and stability. Immobilized bacteria can enhance their physical characteristics, which results in promoting mass transfer of substrate from the environment to the central reaction site. Secondly, Iron oxide NP could facilitate the bacterial bioactivity and metabolite due to their unique surface and quantum size effects. The energy plays a very important role in microbe’s capability in the degradation of organic pollutants. Nitrogenase and hydrogenase are the key enzymes in energy metabolism of PSB (Khusnutdinova et al., 2012; Lo et al., 2012). Due to the presence of [Fe–S] and [Fe–Fe] at the active sites of the nitrogenase and hydrogenase, respectively, an addition of Fe_3_O_4_ NP can improve their enzymatic activities. By coupling the Fe^2+^/Fe^3+^ redox pair with the bacterial oxidation/reduction reactions, the Fe_3_O_4_ NP will speed up the electron transfer rates. Such observations have been reported by Gadhe et al. (2015), in which authors found iron oxide NP promoted biohydrogen recovery from dairy wastewater. Another study was reported that such NP facilitated methanogenesis by enabling direct interspecies electron transfer in syntrophic methane production (Jiang et al., 2013). Our previous investigations (He et al., 2016) have Fe_3_O_4_ NP can promote the growth, metabolism and enzyme activities of bacteria. Moreover, Fe_3_O_4_ can act as extracellular electron acceptors to efficiently scavenge reducing equivalents, thus are speculated to be able to stimulate the growth and metabolisms of PSB (Kato et al., 2012). In addition, a small quantity of iron ions are gradually released into environment by Fe_3_O_4_ NP. Using ICP-OE, we detected ∼10 μg/ml Fe^3+^ in Fe_3_O_4_ NP-saturated wastewater after 3 days (Supplementary Figure 1). The presence of iron ions can stimulate the growth and metabolism of PSB, thus improves the COD removal.

#### Removal of N and P

Our study clearly shows the combination of Fe_3_O_4_/biochar and PSB can enhance the removal of NH_4_^+^ and PO_4_^3-^ as well. N and P are essential nutrients for bacteria growth and they play an important role in substance and energy metabolisms. NH_4_^+^ can be assimilated by almost all PSB to synthesize proteins or other organic biomass species. Phosphorous can be readily accumulated in PSB cells as polyphosphate (poly-P) (Wang et al., 2014). Therefore, it is not unexpected to find PSB-only treatment removes 61% NH_4_^+^ and 63% PO_4_^3-^ (**Figures 5B,C**).

The Fe_3_O_4_/biochar nanocomposite demonstrates a significant enhancement in the PO_4_^3-^ removal (*P* < 0.05) (**Figure 5C**). Not only does the removal efficiency almost reach 100%, but also the time needed for the maximum removal is significantly shortened (*P* < 0.05). More than 92% of phosphates are removed by the nanocomposite within 20 min. The high efficiency of Fe_3_O_4_ NP in phosphate removal is attributed to their larger surface areas and surface hydroxide groups (Lalley et al., 2016). Phosphate ions can be absorbed on the NP’s magnetite surface, followed by diffusion into their interior pores. Electrostatic interaction between the positively charged Fe_3_O_4_ surface and negatively charged phosphate ion plays the major role in the adsorption. In addition, PO_4_^3-^ can replace hydroxyl ions on the NP surface (Chen et al., 2016) and form inner-sphere complexes, including monodentate, bidentate, mono-nuclear or binuclear complexes. Compared to phosphate, the adsorption capacity of Fe_3_O_4_/biochar for NH_4_^+^ is relatively low. This is mostly due to the repulsive electrostatic interaction between Fe_3_O_4_ surfaces and NH_4_^+^. At equilibrium, only about 52% of NH_4_^+^ are removed by Fe_3_O_4_/biochar nanocomposite.

When PSB are immobilized onto Fe_3_O_4_/biochar nanocomposite, the final composite product has an even higher performance in N and P removal (*P* < 0.05). In our experiments, 94% of PO_4_^3-^ and 86% of NH_4_^+^ were removed from the wastewater. This outcome indicates that this Fe_3_O_4_/biochar nanocomposite has a higher NH_4_^+^ removal efficiency than the constructed wetlands methods (∼46% NH_4_^+^) (Wang et al., 2016) and a better PO_4_^3-^ removal efficiency than the active sludge (67–70%) (Vaiopoulou and Aivasidis, 2008; Eldyasti et al., 2011).

The abundances of PSB immobilized onto Fe_3_O_4_/biochar during nutrient removal were quantified by qPCR. As shown in Supplementary Figure 2, the quantity of immobilized PSB increases by ∼10% after the wastewater treatment. This finding suggests that nutrients in wastewater could stimulate the PSB immobilization, an indirect proof for the synergy of PSB and Fe_3_O_4_/biochar. Undoubtedly, the higher amount of immobilized PSB eventually leads to an increase in the nutrients removal elevated consuming quantity of organics, ammonia, and phosphate that can be used for bacterial growth. The growth of cells could be beneficial to the bioremediation of wastewater.

### Reusability of PSB/Fe_3_O_4_/Biochar Composites

Another important property for an ideal wastewater treatment is the recyclability of biocatalyst. Two crucial questions need to be asked: can it be easily recovered, and does the recycled catalyst maintain its activity? The PSB/Fe_3_O_4_/biochar composite excels in both aspects. First, benefited from the magnetism of iron oxide, this composite it can be easily recovered by a magnet bar. Fe_3_O_4_ NP has the magnetic property. Magnetism is a unique physical property that independently helps in water purification by influencing the physical properties of contaminants in water. Adsorption procedure combined with magnetic separation has therefore been used extensively in water treatment and environmental cleanup (Xu et al., 2012). For example, Fe_3_O_4_ hollow nanospheres were shown to be an effective sorbent for red dye (Iram et al., 2010). The saturation magnetization of prepared nanospheres was observed to be 42 emu g^-1^, which was sufficient for magnetic separation with a magnet (critical value at 16.3 emu g^-1^). Secondly, the recycled composite does not lose its activity. We measured the nutrient removal capabilities of PSB/Fe_3_O_4_/biochar composite for up to five recycles (**Figure 5D**). Consistent COD (steadily at 83%) and NH_4_^+^ (86–88%) removal efficiencies are observed throughout these cycles. Although its efficiency in PO_4_^3-^ removal gradually decreases from 94% in cycle-1 to 88% in cycle-5, the PSB/Fe_3_O_4_/biochar composite remains as a very effective PO_4_^3-^ removal agent. As we discussed above, the amount of immobilized PSB increases upon wastewater treatment. Undoubtedly, this is one of the reasons why the composite maintains its high nutrient removal efficiency. Based on these findings, it is clear that PSB/Fe_3_O_4_/biochar composite is an ideal agent for wastewater treatment.

## Conclusion

In this study, we prepared Fe_3_O_4_/biochar nanocomposite by the self-assembly method. The Fe_3_O_4_ were well dispersed on the biochar with little aggregation. Subsequently, PSB were immobilized onto it to produce the final PSB/Fe_3_O_4_/biochar composite product. Our results demonstrate this composite possesses excellent properties for an ideal agent for practical wastewater treatments. Its nutrient (COD, N, and P) removal efficiencies remain high even after five cycles. Its magnetic property enables an easy recovery at the end of each cycle. Besides water treatment, we believe its application may also be expanded to the biodegradation of other hazardous compounds in environment.

## Author Contributions

SH and LY designed and conducted the experiments. JD, YF, and BY analyzed the data. SH, LZ, and LY wrote the paper.

## Conflict of Interest Statement

The authors declare that the research was conducted in the absence of any commercial or financial relationships that could be construed as a potential conflict of interest.
