# Supplementary material for: Bioremediation of Wastewater by Iron Oxide-Biochar Nanocomposites Loaded with Photosynthetic Bacteria
Source: Front Microbiol. 2017 May 23;8:823. doi: 10.3389/fmicb.2017.00823 (PMC5440585; doi:10.3389/fmicb.2017.00823)
Supplement: Supplementary file 1 [file Image_1.PDF]

Supplementary Material

Bioremediation of wastewater by iron oxide-biochar nanocomposites loaded with photosynthetic bacteria

Shiying He[1], Linghao Zhong[2], Jingjing Duan[1], Yanfang Feng[1], Bei Yang[1], and Linzhang Yang[1,*]

*** Correspondence:** Corresponding Author: E-mail: lzyang@issas.ac.cn

1 Supplementary Figure

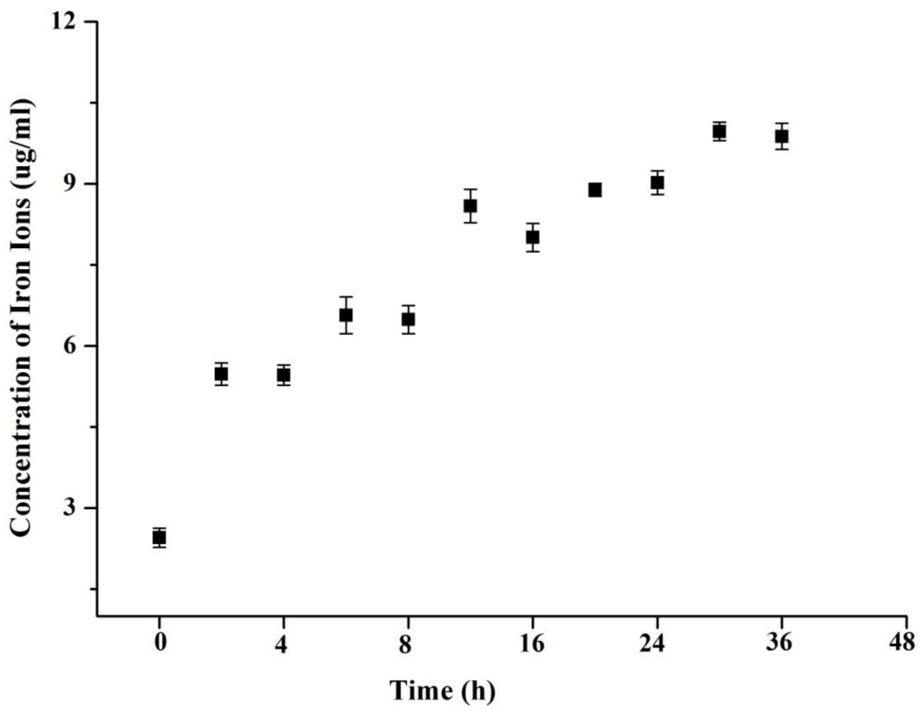

Supplementary Figure 1. The amount of iron ions released into environment by Fe_3O_4 NPs.

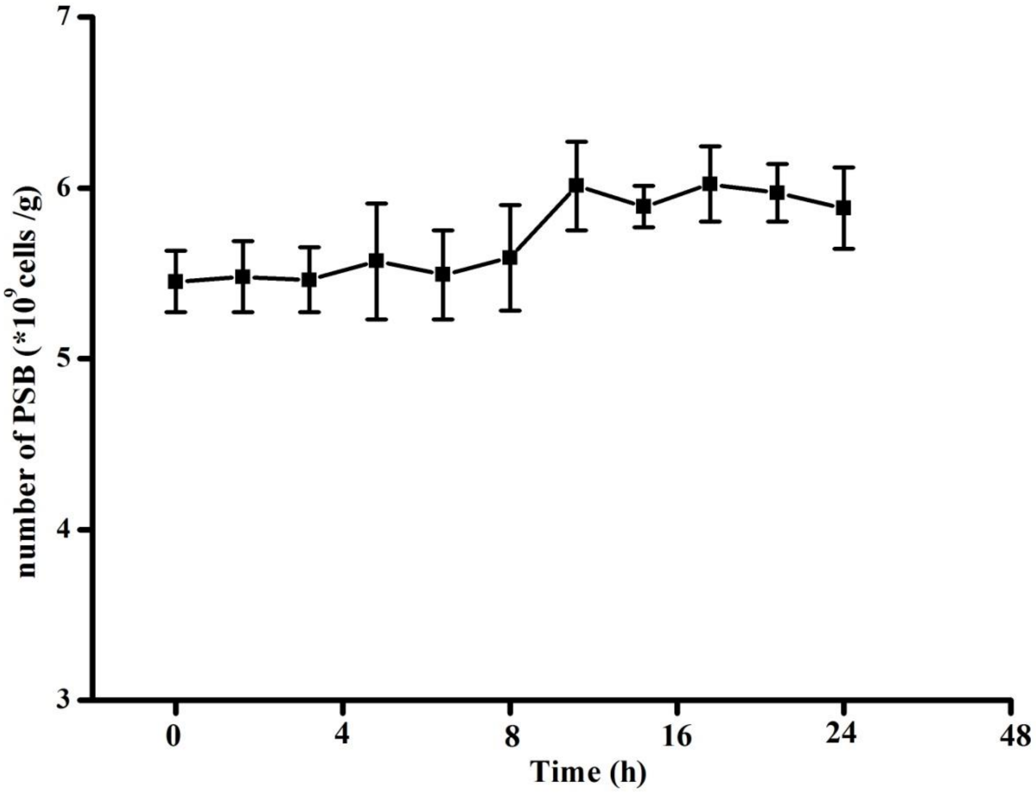

Supplementary Figure 2. The abundances of PSB immobilized on Fe_3O_4/biochar during nutrient removal were quantified by qPCR.
